# Supplementary material for: A fault-intrusion-tolerant system and deadline-aware algorithm for scheduling scientific workflow in the cloud
Source: PeerJ Comput Sci. 2021 Nov 2;7:e747. doi: 10.7717/peerj-cs.747 (PMC8576554; doi:10.7717/peerj-cs.747)
Supplement: Supplemental Information 6 [file peerj-cs-07-747-s006.zip › Myproject1.1/docs/org/cloudbus/cloudsim/network/datacenter/package-summary.html]

org.cloudbus.cloudsim.network.datacenter


JavaScript is disabled on your browser.


- Overview
- Package
- Class
- Use
- Tree
- Deprecated
- Index
- Help

- Prev Package
- Next Package

- Frames
- No Frames

- All Classes

# Package org.cloudbus.cloudsim.network.datacenter

- Class Summary

  | Class | Description |
  | --- | --- |
  |  |  |
  | --- | --- |
  | AggregateSwitch | This class allows to simulate aggregate switch for Datacenter network. |
  | AppCloudlet | AppCloudlet class represents an application which user submit for execution within datacenter. |
  | EdgeSwitch | This class allows to simulate Edge switch for Datacenter network. |
  | HostPacket | HostPacket represents the packet that travels through the virtual network with a Host. |
  | NetDatacenterBroker | NetDatacentreBroker represents a broker acting on behalf of Datacenter provider. |
  | NetworkCloudlet | NetworkCloudlet class extends Cloudlet to support simulation of complex applications. |
  | NetworkCloudletSpaceSharedScheduler | CloudletSchedulerSpaceShared implements a policy of scheduling performed by a virtual machine. |
  | NetworkConstants |  |
  | NetworkDatacenter | NetworkDatacenter class is a Datacenter whose hostList are virtualized and networked. |
  | NetworkHost | NetworkHost class extends Host to support simulation of networked datacenters. |
  | NetworkPacket | NewtorkPacket represents the packet which travel from one server to another. |
  | NetworkVm | NetworkVm class extends Vm to support simulation of networked datacenters. |
  | NetworkVmAllocationPolicy | NetworkVmAllocationPolicy is an VmAllocationPolicy that chooses, as the host for a VM, the host with less PEs in use. |
  | RootSwitch | This class allows to simulate Root switch which connects Datacenter to external network. |
  | Switch |  |
  | TaskStage | Taskstage represents various stages a networkCloudlet can have during execution. |
  | WorkflowApp | WorkflowApp is an example of AppCloudlet having three communicating tasks. |

- Overview
- Package
- Class
- Use
- Tree
- Deprecated
- Index
- Help

- Prev Package
- Next Package

- Frames
- No Frames

- All Classes
